# Severe esophagitis induced by antituberculosis drugs: a case report

**DOI:** 10.1590/S1678-9946202466002

**Published:** 2024-01-05

**Authors:** Roxana Flores Mamani, Franciele Moreira Silva, Marcos Vidal de Lima-Júnior, Juliana Paitach de Oliveira Lima, Vitor Montez Vianna, Rivelino Trindade de Azevedo, Billy McBenedict, Ezequias Batista Martins

**Affiliations:** 1 Hospital Municipal Ronaldo Gazolla, Rio de Janeiro, Rio de Janeiro, Brazil; 2 Universidade Federal Fluminense, Faculdade de Medicina, Niterói, Rio de Janeiro, Brazil

**Keywords:** Tuberculosis, Treatment, Ulcerative esophagitis, Serious adverse reaction

## Abstract

Tuberculosis stands as one of humanity’s oldest afflictions, intrinsically intertwined with social disparities. This formidable disease spares no age group and remains the prevailing cause of infection-induced mortality worldwide, particularly in developing nations. We present a case of a 56-year-old woman with diabetes who was diagnosed with Pulmonary Tuberculosis. After receiving antituberculosis drugs as part of her treatment, she experienced a range of systemic manifestations and suffered from severe ulcerative esophagitis. This adverse reaction led to uncontrollable gastrointestinal intolerance, tragically resulting in her untimely demise. The incident underscores the potential seriousness of adverse reactions that can arise from tuberculosis treatment medications.

## INTRODUCTION

Tuberculosis remains one of the main causes of morbidity and mortality in the world. It is crucial to promptly diagnose adverse reactions to antituberculosis drugs. Adverse reactions most commonly associated with the use of antituberculosis drugs include hepatitis, chemical reactions, gastric intolerance, hematologic disorders, and severe renal disorders, which demand early detection^1^.

Adverse reactions to antituberculosis drugs can be “minor” (discontinuation of antituberculosis drugs is not necessary) or “major” (discontinuation of treatment is required). The frequency of “major” adverse reactions ranges from 3% to 8% and is associated with risk factors such as age (from the fourth decade of life), alcohol dependence (daily dose > 80g), malnutrition, prior liver disease, and AIDS^2^.

The most frequent adverse reactions to the standard regimen of treatment with Rifampicin, Isoniazid, Pyrazinamide, and Ethambutol (RHZE) include choluria (occurs universally), gastric intolerance (40%), skin disorders (20%), jaundice (15%), and joint pain (4%)^2-4^. Following the administration of RHZE, individuals may experience digestive intolerance, characterized by symptoms such as nausea and vomiting, as well as epigastric pain^2^.

Among first-line drugs, Rifampicin usually causes anorexia, nausea, vomiting, abdominal discomfort, and diarrhea. In some cases, these side effects might require replacing Rifampicin with alternative drugs^1^. Symptoms may appear at any time during treatment. More serious adverse reactions are rarely observed, such as: eosinophilic colitis^5^, pseudomembranous colitis^6^, drug-induced esophagitis^1^, and upper gastrointestinal bleeding^7^.

In this work, a case of severe ulcerative esophagitis with upper digestive hemorrhage after antituberculosis drugs use is reported. Rifampicin was suspected as the main drug responsible for inducing the aforementioned adverse reaction.

## CASE REPORT

A 56-year-old White female, general service assistant presented with hypertension and diabetes as a comorbidity for which she was on regular treatment with metformin, glybenclamide, and losartan. On January 12, 2023, she was diagnosed with pulmonary tuberculosis, confirmed by a positive acid-fast bacilli (AFB) sputum smear. On the same day, the patient weighing 49kg commenced treatment with Rifampicin (450mg/day), Isoniazid (225mg/day), Pyrazinamide (1,200mg/day), and Ethambutol (825mg/day).

During the first week of treatment, the patient experienced severe gastric intolerance, characterized by epigastric pain, nausea, and vomiting. To address this issue, she was recommended to take the medication after breakfast, and was prescribed antiemetic drugs (Bromopride, 10 mg, three times per day) and gastric protectors (Omeprazole, 20mg, once per day). Despite these measures, her gastric intolerance escalated, eventually leading to diabetes decompensation with symptoms of dehydration, malnutrition, and hyperglycemia. Nevertheless, she committed to keep taking the medication as prescribed.

On March 6, 2023 (54 days after the start of treatment) the patient developed severe gastrointestinal disorders (diarrhea, nausea, and unrelenting vomiting), hemodynamic decompensation, and signs of pre-shock, requiring hospitalization. Proton pump inhibitors (Omeprazole, 40 mg, twice per day) and potent antiemetics (ondansetron, 8 mg, three times per day) were administered intravenously, with symptom alleviation. Laboratory tests upon admission confirmed severe hyperglycemia, acute kidney injury, and severe leukocytosis. Treatment with cephepime (3000mg/day) was initiated due to the possibility of a bacterial infection. The RHZE scheme was suspended and intensive care was instituted. The blood sample collected before antibiotic therapy showed negative results on the culture test. Diagnostic tests for HIV, SARS-CoV-2, and Hepatitis B/C were negative. AFB in sputum was negative. Chest tomography (March 10, 2023) showed apical pleural thickening, central acinar nodules, and small bilateral pleural effusion.

**Figure 1:**
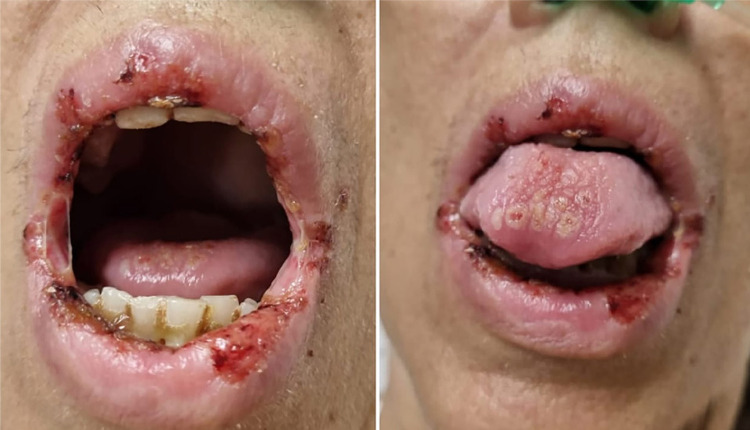
Ulcerative lesions in the patient’s oral cavity, on hospital admission.

Figure 1 shows ulcerated lesions in the patient’s oral cavity, as observed on the day of hospitalization.

**Figure 2:**
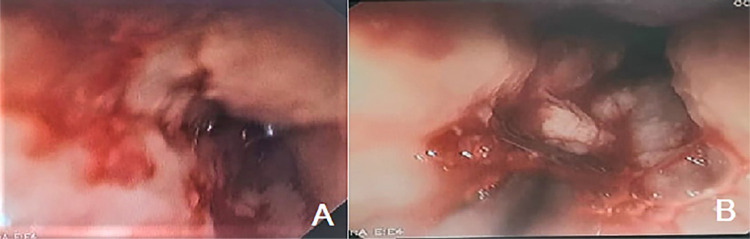
Digestive endoscopy: A) Esophagus with confluent elevated white plaques adhered to the wall, along with hyperemia and friable membranes; B) evidence of severe, circumferential, fibrin-covered, ulcerative esophagitis bleeding with signs of mucositis.

On March 10, 2023 (fourth day of hospitalization), the patient was hemodynamically stable and the laboratory parameters improved, allowing the reintroduction of antituberculosis drugs. On March 13, 2023 (three days after the reintroduction of RHZE), the patient had intense nausea, severe epigastric pain, uncontrollable vomiting, hematemesis, disorientation, and progressive worsening of renal function. On the same day, she underwent orotracheal intubation and hemodialysis. On March 16, 2023, the patient was diagnosed with severe anemia (Haemoglobin: 5.7 mg/dL – Haematocrit 15.7%), and, on the same day, an upper digestive endoscopy examination diagnosed severe ulcerative esophagitis (Figure 2). Skin lesions were not observed at any time, reducing the possibility of a serious hypersensitivity reaction affecting the esophageal and gastric mucosa. Antituberculosis drugs were discontinued and the patient was extubated on March 20, 2023, after clinical and laboratory improvement.

Subsequent upper digestive endoscopy examination (March 24, 2023) showed whitish plaques in the esophagus that are compatible with esophageal candidiasis (Figure 2). The patient was treated with fluconazole (200mg/day) for seven days. On March 26, 2023, RHZE drugs were reintroduced (same doses) and unrestrained nausea and vomiting manifested following drug administration. On March 28, 2023, alternative drugs for the treatment of tuberculosis were recommended, with amikacin (750 mg/day) and levofloxacin (500 mg/day). On March 29, 2023, the patient developed high fever, severe dyspnea, and neurological disorders, requiring orotracheal intubation. On April 1, 2023 (27th day of hospitalization), the patient died from septic shock resulting from a bacterial infection (probable vascular catheter focus). Table 1 shows the results of the main laboratory tests performed during the patient’s hospitalization.

**Table 1:** Laboratory tests performed during hospitalization.

	Mar 06, 2023	Mar 10, 2023	Mar 16, 2023	Mar 29, 2023
Haemoglobin(g/dL)	11.6	10.2	5.7	7.9
Haematocrit (%)	33.2	27.2	15.7	21.6
White blood cells (x10^3^/µL)	23,000	7,770	9,620	21,030
Bands cells (%)	9	4	2	6
Platelets (x10^3^/µL)	269,000	215,000	193,000	181,000
Alanine aminotransferase (IU/L)	32	22	28	34
Alkaline phosphatase (IU/L)	55.5	31	40	52
International normalized ratio	1.31	1.4	1.29	1.17
Blood urea nitrogen (mg/dL)	145	115	140	50
Creatinine (mg/dL)	3.20	3.28	7.29	1.9
Potassium (mEq/L)	6.4	3.5	4.4	4.6
C-Reactive protein (mg/dL)	1.20	1.30	4.97	6.40
Glucose (mmol/L)	> 500	120	84	260

## DISCUSSION

An important adverse reaction following the administration of antituberculosis drugs was described in this manuscript. Undoubtedly, the gastrointestinal symptoms prevented the regular use of medication, hindering adequate treatment of tuberculosis.

In the co-occurrence of tuberculosis and diabetes, a lower serum concentration of antituberculosis drugs and a greater risk of drug toxicity is observed. Diabetes slows down the immune response, predisposing an individual to increased relapses and increasing the risk of resistance^8^. Therefore, strict monitoring of blood glucose during tuberculosis treatment is very important^9^. The presence of adverse reactions to the treatment must be early identified and concomitant treatment of other diseases, such as diabetes, must be rigorously adequate. It is very important to identify all comorbidities to ensure proper management of possible complications.

The World Health Organization describes some adverse reactions to antituberculosis drugs^10^. Among the most frequently reported adverse reactions, gastrointestinal disorders are frequent (40%) and, in most cases, treatment can proceed^2^. Symptoms such as nausea, vomiting, and abdominal pain can be controlled by changing the time of drug intake and adding auxiliary medications. All four drugs used as a first-line treatment for tuberculosis can be linked to the aforementioned symptoms, including upper gastrointestinal bleeding. However, rifampicin is the most commonly associated with these symptoms^1,5-7^. We highlight that the observed severe gastrointestinal symptoms hindered the use of antituberculosis drugs. Thus, the hypothesis suggesting rifampicin as the trigger for ulcerative esophagitis remains consistent.

An important review study showed that esophageal tuberculosis can be manifested by dysphagia, odynophagia, chest pain, and ulcerated lesions in the esophagus^11^. In the case presented, the gastrointestinal symptoms were directly related to antituberculosis drugs, with alleviation of symptoms during drug discontinuation. For these reasons, it was not suspected to be a case of esophageal tuberculosis.

To the best of our knowledge, no reports have focused on the manifestation of severe ulcerative esophagitis, secondary to using RHZE in immunocompetent patients^12^. Inflammation of the esophageal mucosa resulted from secondary gastroesophageal reflux, culminating in upper digestive hemorrhage^13^. This manuscript reports a case of tuberculosis with persisting gastrointestinal symptoms mostly triggered by antituberculosis drugs, evinced by the repeated occurrence of these symptoms whenever the patient took antituberculosis drugs. Despite applying supportive measures, these symptoms showed no improvement, suggesting that diabetic patients should be included in the category of immunocompromised individuals.

## CONCLUSIONS

Early identification of severe adverse reactions following the administration of antituberculosis drugs is crucial to evaluate the necessity of switching from first-choice drugs to alternative ones. Notably, patients with type 2 diabetes mellitus, despite receiving regular and effective treatment, are at a higher risk of experiencing clinical decompensation, and thus it is imperative to consider drug interactions. Early referral of patients with gastric intolerance to specialized services is recommended to facilitate improved monitoring of their condition.
